# Incisional Site Metastasis in a Patient with Cervical Carcinoma: A Case Report and Review of the Literature

**DOI:** 10.1155/2012/593732

**Published:** 2012-11-26

**Authors:** C. Iavazzo, K. Madhuri, A. Tailor, S. Butler-Manuel

**Affiliations:** Gynaecological Oncology Department, Royal Surrey County Hospital, Guildford, Surrey GU2 7XX, UK

## Abstract

Abdominal wall metastasis either incisional, drain, or port is rather rare in patients treated for cervical carcinoma. We present a case of a patient who underwent an abdominal radical hysterectomy for a moderately differentiated cervical adenocarcinoma stage Ib1 and presented an incisional site metastasis 36 months after her operation. Moreover, we performed a literature search for abdominal wall metastases after radical hysterectomy for cervical cancer, and we present a table of the relative case reports. After our literature search, we clarified that the median time of recurrence was 14 months (range 1.5 month to 45 months). Thirty-three out of 42, 8/42, and 1/42 were squamous, adeno-, and adenosquamous carcinomas, respectively. Wide excision was performed in 30/37 cases of which we have information regarding the treatment option, while 11/37 and 13/37 underwent radiotherapy and chemotherapy, respectively. The possible mechanism of such a metastasis as well as the treatment options is discussed.

## 1. Introduction

 Common extrapelvic metastases of cervical carcinoma are mainly lymphatic, lungs, liver, and bone. Rarely (0.1–1.3%), metastatic recurrence of squamous cervical carcinoma could be identified in the abdominal wall especially in sites of incision [1]. Metastatic skin incisional cancers are usually presenting in cases of adenocarcinomas of the ovaries, colon, pancreas, kidneys, or bladder [2]. Based on the common metastasis of such adenocarcinomas, one could understand the possible mechanism of cervical adenocarcinomas; however, it is difficult to clarify the mechanism in squamous cell carcinomas.

We present a case of an incisional site metastasis three years after radical hysterectomy for cervical cancer, as well as a review of the current literature in the field. For this reason, a relevant search with the terms cervical cancer, metastasis, abdominal wall, drain-site, port-site, adenocarcinoma, and/or squamous cell has been performed in Pubmed.

## 2. Case

A 46-year-old morbidly obese patient underwent an abdominal radical hysterectomy for a moderately differentiated cervical adenocarcinoma stage Ib1 on 2007. No adjuvant radiotherapy was necessary at that moment. Two years later, she presented with incisional hernia, for which she underwent a repair. During this operation, her bowel was injured, and the colonic perforation led to faecal peritonitis. For this reason, Hartman's procedure was performed as an emergency and prolonged ITU admission was essential. Three years after her first operation, the patient presented to our clinic with a recurrence of adenocarcinoma in the anterior abdominal wall at the site of the hernia and intimately related to the stoma measuring 7 cm. The C/T-guided biopsy revealed cores of fibrofatty connective tissue with stromal desmoplasia and admixed sheets of foam macrophages. Focally, the cores showed a lining of glandular epithelium with marked nuclear pleomorphism and multiple mitoses consistent with metastatic adenocarcinoma. As the patient was cisplatin naïve, she completed six cycles of chemotherapy (cisplatin and topotecan). For 6 months, she had a good response to palliative treatment as there has been marked reduction in size down to 4.3 cm. That was the time of a new relapse and for this reason, a wide excision of the recurrence in the anterior abdominal wall as well as reverse of the stoma was performed, followed by use of a synthetic mesh to cover the defect. An 8 cm tumor was found in the left lower part of the rectus abdominis muscles. It was infiltrating the sheath but not involving any intra-abdominal structures. Histology identified adenocarcinoma identical to her primary cervical cancer.

## 3. Discussion

Incisional site metastasis is an extremely rare complication of cervical carcinoma especially of the squamous type. To clarify the rarity of such metastases, Zivanovic et al. showed that two cases out of 1694 patients with a malignant gynaecological condition operated laparoscopically had a port-site metastasis compared with 15 patients of ovarian cancer in the same series [23]. More specifically, Chen et al. in a prospective study including the followup of 295 patients with cervical carcinoma presented only one patient with trocar incision site metastasis [21]. Similarly, in a large retrospective study including 921 patients with cervical carcinoma, only 0.43% presented with port-site metastasis [20]. For this reason, evidence-based conclusions could be hardly raised as they are based on case reports. This is why we present on Table 1 the characteristics of the patients with such a recurrence. 

The actual incidence of incisional site metastasis might be higher than identified in this paper (31 Cases) caused by the lack on reporting them. It should be notified that 19 cases were identified after laparotomies, 21 cases after laparoscopic procedures, while only two cases were found in a robot-assisted radical hysterectomy [29, 30], as this is a rather new technique. It could be raised, however, that laparoscopic and robotic operations for cervical carcinomas could lead to iatrogenic metastases at the port sites on a similar mechanism. In robotic cases, the pressure required for exposure is smaller compared to laparoscopy due to the mechanical lift of the robot [31].

After our literature search, we clarified that the median time of recurrence was 14 months (range 1.5 month to 45 months). Thirty-three out of 42, 8/42, and 1/42 were squamous, adeno-, and adenosquamous carcinomas, respectively. Wide excision was performed in 30/37 cases of which we have information regarding the treatment option, while 11/37 and 13/37 underwent radiotherapy and chemotherapy, respectively.

Our results could be compared to a case series where 12 patients were identified with port-site metastasis after laparoscopy for cervical cancer with a median age of 44 years (range 31–74 years). Eighty per cent of them were squamous carcinomas, and 67% were diagnosed in an early stage. The median time for diagnosis was 5 months (1.5–19 months). Regarding their management, 78% of the tumors were excised, chemotherapy alone was used in 3/12 patients, combination of chemoradiation in 3/12, while radiotherapy in 1/12 patients [32].

The potential risk factors of such metastases could be divided into four groups (Table 2): the first group includes all the parameters relative to the patient such as local immunoreactions, wound hypoxia, and acidosis leading to angiogenesis and hematogeneous spread around umbilicus. For example, in an animal model the use of an intraperitoneal endotoxin as an immune enhancer led to less common tumor growth and port-site metastasis [33]. The second group includes the disease itself (advanced disease, adenocarcinoma cell type, peritoneal carcinomatosis, and lymph node disease). According to Imachi et al. the incidence of skin metastases squamous cell carcinoma is 0.9% and adenocarcinoma 5.8%. Moreover, there is a 6-fold greater risk for stage IV compared to I [34]. It should also be mentioned, however, that metastases can occur even in cases of microscopic disease [11]. Regarding the seed and soil theory, it should be noted that cells can spread through the body via blood or lymph vessels. Most cells do not survive while a small amount develop metastasis depending on tissue microenvironment and tumor type. The third group includes parameters relative to the surgical technique (mechanical port irrigation, not use of endobags, trocar size direct implantation by instruments or gloves), while the fourth group those of the laparoscopic environment (pneumoperitoneum and especially use of carbon dioxide). Iwanaka et al. showed that there is no difference in the rate of port site metastases when gasless laparoscopy is used [35]. Protective measures could be trocar fixation, prevention of gas leaks, slow deflation of peritoneum, and povidone-iodine rinsing of instruments and trocars before their removal [20]. Another measure for minimizing port-metastasis could be lavage of peritoneal cavity with heparin, povidone-iodine, methotrexate, and/or normal saline [36]. 

Biopsy is necessary to prove the origin of the metastasis. Moreover, immunohistochemical findings such as strong CD31 positivity could help to identify the relativity of the metastasis to the primary lesion [20]. 

It should be noticed that it is very difficult to compare laparotomy to laparoscopy or robotic technique regarding incisional metastases. There is strong heterogeneity of the findings. Regarding the possible mechanism of metastasis to the abdominal wall, several theories have been raised. Kadar believed that dissemination results from the enhancement of tumor growth characteristic of early healing tissues and so metastasis could be prevented by appropriate postoperative treatment [7]. Cell dilution assays have shown that fewer tumor cells are necessary to induce tumorogenesis in skin incisions compared to unwounded skin [37]. Overmanipulation of the disease during laparoscopic procedures may result in tumor spillage, intraperitoneal dissemination, and wound contamination. It should be mentioned that port-site metastasis on 10 mm trocar sites are rarely found [7]. Martínez-Palones et al. tried to explain the mechanism by the peritoneal increase in microvessel density as well as the strong CD31 positivity which both suggested angiogenesis [20]. Gregor et al. suggested that surgeons should reduce mechanical irritation of port sites and spillage of tumor cells [16]. Paolucci et al., however, believe that an intact surgical specimen and the use of a plastic retrieval bag does not minimize the cancer risk [38]. The most possible mechanism, however, should be the so-called “chimney effect” (leakage of CO_2_ along trocars) caused by pneumoperitoneum, meaning that cells dislodged at the time of cervical manipulation may pass through the fallopian tubes and implant in the port sites [12]. In open laparotomy, there is no “contaminating seeding” passage of the tumor through a narrow incision.

In our case, during the second operation for hernia repair, there was no intraperitoneal disease. Colonic perforation as well as the following peritonitis could be the first trigger of this sequence of events causing spillage of tumor cells.

Careful and close followup including examination and imaging with special attention to incisional, port, or drain sites is proposed to early identify such a recurrence. Treatment of such recurrences remains palliative and includes chemotherapy, radiotherapy, or wide surgical excision. For example, reconstruction of the abdominal wall with a latissimus dorsi musculocutaneous flap as well as mesh use are proposed in the literature [4]. Platin-based chemotherapy is usually recommended, while schemes with fluorouracil or topotecan have also been used. Although treatment should be individualized, it seems that wide excision in combination with chemotherapy could be the best treatment option.

It is difficult to clarify the prognosis of such a recurrence as patients could die because of their extensive disease. Prognosis in such cases is usually poor because of the systematic contamination of the disease,; however, survival could reach even 4.5 years [13]. Ramirez et al. showed that in a median follow-up period of 12 months, 63% of patients died of disease [32].

Although, some could declare that incisional site metastasis and abdominal wall metastasis should not be confused, the possible mechanism of metastasis might be relatively similar. For this reason, we added possible hypotheses—pathways of such rare metastases. Abdominal metastasis after radical surgery for squamous cervical carcinoma is a rare entity; however followup and treatment should be further clarified. A multicenter analysis is proposed in order to clarify the presentation and management of such rare entities.

## Figures and Tables

**Table 1:** Abdominal metastasis after radical surgery for cervical carcinoma.

Author/year/age of patient	Histological type	Stage	Type of operation	Postoperative radiotherapy	Site of metastasis	Time of recurrence	Treatment
Singh and Salwan [3]/1976/53 y.o.	Squamous	IV	Total pelvic exenteration	No	Urinary conduit stoma	12 months	Wide excision, radiotherapy

Neven et al. [4]/1993/49 y.o.	Squamous	IB	Abdominal radical hysterectomy	Yes	Abdominal wall	24 months	Wide excision

Copas et al. [5]/1995/46 y.o.	Squamous	IIA	Retroperitoneal pelvic and paraaortic lymphadenectomy	Yes	Drain site	7 months	Wide excision, radiotherapy

Naumann and Spencer [6]/1997/41 y.o.	Squamous	IIIB	Laparoscopically guided placement of Syed needle	Yes	umbilical	5 months	Wide excision

Kadar [7]/1997/64 y.o.	Squamous	IIB	Laparoscopic lymphadenectomy	Yes	port		Wide excision

Kadar [7]/1997/41 y.o.	Squamous	IIB	Laparoscopic lymphadenectomy	Yes	port		Wide excision

Wang et al. [8]/1997	Squamous	IB	Laparoscopically assisted vaginal hysterectomy		port	2 months	

Lavie et al. [9]/1999/48 y.o.	Adenocarcinoma	IA1	Laparoscopically assisted vaginal hysterectomy	No	port	9 months	Wide excision

Carvalho et al. [10]/1999/33 y.o.	Squamous	IB2	Laparoscopic lymphadenectomy	Yes	port	1.5 months	

Lane and Tay [11]/1999/58 y.o.	Adenosquamous	IB1	Laparoscopic lyphadenectomy	Yes	Port	10 months	Wide excision

Kohlberger et al. [12]/2000/31 y.o.	Squamous	IB	Laparoscopic radical hysterectomy	No	Suprapubic port	19 months	Wide excision, radiotherapy

Doret et al. [13]/2000	Squamous	IIB	Laparoscopic radical hysterectomy		Port		

Agostini et al. [14]/2003/6 y.o.	Squamous	IIB	Laparoscopic lymphadenectomy	Yes	port	8 months	Wide excision

Tjalma et al. [15]/2001/74 y.o.	Squamous	IIIB	Laparoscopic retroperitoneal paraortic lympadenectomy	Yes	umbilical	15 months	Chemotherapy

Gregor et al. [16]/2001/31 y.o.	Squamous	IIB	Laparoscopic lymphadenectomy	Yes	port	3 months	Wide excision

Behtash et al. [17]/2002/44 y.o.	Squamous	IIA	Radical hysterectomy	Yes	drain	9 months	Chemotherapy, radiotherapy

Liro et al. [18]/2002	Squamous	IIA	Radical hysterectomy	Yes	Abdominal wall	6 months	Excision, chemotherapy

Picone et al. [19]/2003/37 y.o.	Adenocarcinoma	IIB	Laparoscopic ovarian transposition	Yes	port	6 months	chemotherapy

Martínez-Palones et al. [20]/2005/9 y.o.	Adenocarcinoma	IIIB	Laparoscopic retroperitoneal paraaortic lymphadenectomy	Yes	umbilical	7 months	Wide excision

Srivastava et al. [2]/2005/35 y.o.	Squamous	IIA	Radical hysterectomy	Yes	Incisional site	3.5 years	Wide excision, chemotherapy

Chen et al. [21]/2008	Squamous		Laparoscopic radical hysterectomy		Port		Wide excision

Iavazzo et al. [22]/2008/24 y.o.	Squamous	IIA	Radical hysterectomy	Yes	drain	14 months	Excision, radiotherapy

Zivanovic et al. [23]/2008	Squamous		Laparoscopy		port		

Zivanovic et al. [23]/2008	Squamous		Laparoscopy		port		

Park et al. [24]/2008/45 y.o.	Adenocarcinoma	IIB	Laparoscopic retroperitoneal paraortic lympadenectomy	Yes	port	4 months	chemotherapy
Ding et al. [25]/2008/45 y.o.	Squamous	IB	Radical hysterectomy		incisional	2.5 years	Wide excision, chemotherapy, radiotherapy

Kim et al. [26]/2008/64 y.o.	Squamous	IB2	Radical hysterectomy	Yes	Abdominal wall	6 months	Chemotherapy, radiotherapy

Yenen et al. [27]/2009/42 y.o.	Squamous	IIB	Laparoscopic retroperitoneal paraortic lympadenectomy	Yes	port	6 months	Wide excision, chemotherapy

van den Tillaart et al. [28]/2010/63 y.o.	Squamous	IIA	Radical hysterectomy	Yes	Abdominal	27 months	Wide excision

van den Tillaart et al. [28]/2010/29 y.o.	Squamous	IIB	Radical hysterectomy	Yes	Abdominal	2 months	Wide excision

van den Tillaart et al. [28]/2010/34 y.o.	Squamous	IIB	Radical hysterectomy	Yes	Abdominal	4 months	Wide excision

van den Tillaart et al. [28]/2010/35 y.o.	Squamous	IB2	Radical hysterectomy	Yes	Abdominal	21 months	Wide excision

van den Tillaart et al. [28]/2010/35 y.o.	Squamous	IB1	Radical hysterectomy	No	Abdominal	11 months	Wide excision, radiotherapy

van den Tillaart et al. [28]/2010/61 y.o.	Adenocarcinoma	IB1	Radical hysterectomy	No	Abdominal	45 months	Wide excision

van den Tillaart et al. [28]/2010/ 65 y.o.	Squamous	IB1	Radical hysterectomy	Yes	Abdominal	10 months	chemotherapy

van den Tillaart et al. [28]/2010/41 y.o.	Squamous	IB2	Radical hysterectomy	Yes	Abdominal	14 months	Chemotherapy

van den Tillaart et al. [28]/2010/44 y.o.	Squamous	IB2	Radical hysterectomy	No	Abdominal	6 months	Wide excision, radiotherapy

van den Tillaart et al. [28]/2010/ 32 y.o.	Adenocarcinoma	IB2	Radical hysterectomy	No	Abdominal	33 months	Wide excision

van den Tillaart et al. [28]/2010/26 y.o.	Squamous	IB1	Radical hysterectomy	Yes		5 months	Wide excision, radiotherapy

Sert [29]/2010/60 y.o.	Adenocarcinoma	IB1	Robotic-assisted radical hysterectomy	No	port	18 months	Wide excision, chemotherapy, radiotherapy

Boiles and Borowsky [30]/2012/35 y.o.	Squamous	IB2	Robotic-assisted radical hysterectomy	Yes	port	5 months	Wide excision

RSCH/2012/46 y.o.	Adenocarcinoma	IB1	Radical hysterectomy	No	incisional	3 years	Chemotherapy, wide excision

**Table 2:** Potential risk factors of abdominal metastasis.

(a) Patient
(i) Local immunoreactions
(ii) Wound hypoxia and acidosis leading to angiogenesis
(iii) Hematogeneous spread around umbilicus
(iv) Inflammation oncotaxis

(b) Disease
(i) Advanced disease
(ii) Adenocarcinoma cell type
(iii) Peritoneal carcinomatosis
(iv) Lymph node disease
(v) Seed and soil theory

(c) Surgical technique
(i) Mechanical port irrigation
(ii) No use of endobags
(iii) Trocar size
(iv) Direct implantation by instruments or gloves

(d) Laparoscopic environment
(i) Use of carbon-dioxide
(ii) Pneumoperitoneum
